# The effect of unhealthy lifestyle on the pathogenesis of sick sinus syndrome: A life-guiding review

**DOI:** 10.1097/MD.0000000000039996

**Published:** 2024-10-25

**Authors:** Xing Chang, Qin Zhang, Xiangyi Pu, Jinfeng Liu, Yanli Wang, Xuanke Guan, Qiaomin Wu, Siyuan Zhou, Zhiming Liu, Ruxiu Liu

**Affiliations:** a Guang’anmen Hospital, Chinese Academy of Traditional Chinese Medicine, Beijing, China.

**Keywords:** lifestyle, pathogenesis, prevention, sick sinus syndrome

## Abstract

Sick sinus syndrome (SSS), also known as sinoatrial node dysfunction, has been widely concerned by the medical community. The incidence rate of SSS is increasingly, which poses a great threat to public health. Through decades of repeated research in the medical field, great progress has been made in the pathogenesis of SSS and the interaction mechanism between SSS and other cardiovascular diseases. In this paper, we pay special attention to the mental stimulation factors under various pressures such as society and work, and the influence of smoking, drinking, and unhealthy diet on the pathogenesis of SSS. It also explains the mechanism of negative factors in the pathogenesis of SSS. These unhealthy lifestyle will lead to the occurrence of sinoatrial node disease and arrhythmia, and then induce SSS. Therefore, in the premise of increasing incidence rate of SSS and difficult to cure, how to avoid these harmful factors and ensure a healthy lifestyle is extremely important for preventing and treating SSS. This study also has guiding significance for the daily life of high-risk population of SSS and reducing the mortality of SSS patients.

## 1. Introduction

Sick sinus syndrome (SSS), also known as sinoatrial node dysfunction. It is an arrhythmia syndrome characterized by heart failure to perform pacing function due to the pathological changes of sinoatrial node and its adjacent tissues.^[1]^ The formation of abnormal beating of the heart and the failure of normal beating of the heart belong to the category of sinoatrial node dysfunction.^[2–5]^ SSS patients will have symptoms such as sinus bradycardia, sinus arrest, alternating arrhythmia, and tachyarrhythmia. Some patients will also have severe syncope.^[6,7]^ This disease is one of the most difficult cardiovascular diseases (CVDs) in clinic. It is mainly diagnosed by ECG, in-hospital monitoring or 24-hour Holter monitoring.^[8,9]^ According to the epidemiological survey, the onset time of SSS tends to be aging, and the main age range of SSS is about 20 to 90 years old, the average age is about 68 years old, which usually occurs in the elderly. According to statistics, there is one SSS patient in every 600 heart disease patients over 65 years old. The incidence rate of SSS is about 0.17% and increases with age. The 5-year survival rate is only 62% to 65%.^[1,10,11]^ In clinical treatment, implantation of permanent pacemaker is the best choice to treat SSS. Pacemaker implantation can effectively reduce the symptoms of syncope, arrhythmia, and improve the quality of life of SSS patients, but it will not reduce the mortality of SSS patients.^[2]^ The sharp increase in the number of patients with SSS and the problem that they cannot be completely cured is one of the biggest challenges facing the medical community.

The etiology and pathogenesis of SSS are complex. Iatrogenic factors such as ischemia and anoxia accompanied by various CVDs and improper drug treatment have been identified as the main risk of increasing the prevalence of SSS.^[12]^ The primary cause of the increased frequency of SSS in older adults is sinus node fibrosis, which is brought on by tissue deterioration in the sinus nodes.^[13–15]^ The process of sinus node fibrosis is accelerated as the heart ages because the fibrous matrix gradually replaces the tissue found in the sinus node and the surrounding myocardial tissue. The pathophysiology of SSS is caused by the process of sinus node fibrosis, which also has an impact on the sinus node’s conduction system.^[16]^ Research findings of the etiology of sick sinus node syndrome have gotten better in the past few years.^[17,18]^ The participants with the healthiest lives had the lowest risk of all-cause mortality, according to a meta-analysis incorporating 142 research. On the other hand, detrimental lifestyle choices (such as smoking, drinking alcohol, engaging in physical activity, eating poorly, and being overweight or obese) markedly raised the risk of CVD and death.^[19]^ This finding holds true for populations who come from various socioeconomic backgrounds, nationalities, and continents.

In addition to the negative effects of unhealthy lifestyles like smoking, binge drinking, having unpredictable work schedules, and leading sedentary lives, young people also experience hypertension, disorders of the neurohormonal secretion system, and ion channel regulation system as a result of psychological decline brought on by various stresses. The most plausible central mechanism that combines all risk variables is the crosstalk among these 3 processes. Research has indicated that low levels of calcium are linked to high blood pressure (BP) and heart problems, and that taking supplements of both calcium and vitamin D together can lower diastolic blood pressure.^[20]^ All unhealthy lifestyles and the effects of stress result in intrinsic pathological mechanisms that lead to secondary alterations such as hypertension, oxidative stress, inflammation, myocardial fibrosis, and so forth. These mechanisms include abnormalities in the renal–angiotensin–aldosterone system, dysfunctions in sympathetic–parasympathetic neuromodulation, dysfunctions in the hypothalamic–pituitary–thyroid and other neuroendocrine axes, and disruptions in calcium-ion homeostasis. The majority of patients with SSS have clinical symptoms such as sinus bradycardia, sinus arrest, atrial flutter, and atrial fibrillation. If these symptoms are not treated, the incidence of ventricular tachycardia or fibrillation will increase, which greatly increases the risk of sudden cardiac death.^[21]^ The primary goal of this study was to examine how abnormalities in psychological state caused by various stressors, with a common poor lifestyle, moderated the effects of SSS development.

## 2. Mental factors

With the continuous progress of society and the rapid development of economy, people’s work pressure and life pressure are increasingly. Different kinds of stressors include physical pressure, social pressure, cultural pressure, psychological pressure and so on. Different kinds of stressors can lead to anxiety, depression, fear, anger, indifference, tension, sadness, and other adverse mental factors. Studies have shown that excessive anxiety, depression, fear, anger, indifference, tension, and sadness are closely related to CVD.^[22–27]^Although the relationship between mental factors and the pathogenesis of SSS has not been elucidated, various negative mental factors should also be considered as risk factors of SSS.^[28]^ Psychological trauma caused by daily work stress and family life stress increases the morbidity and mortality of various CVDs including SSS.^[29,30]^A prospective meta-analysis observation study has reported that long-term social isolation and loneliness increased the risk of CVD by 50%.^[31]^ Loneliness and isolation were substantially linked to essential hypertension, arteriosclerosis heart disease, myocardial infarction, and angina in a two-sample Mendel randomized trial.^[32]^Different stress patterns from childhood to adulthood have been found to have an impact on adult cardiometabolic risk. An 18-year prospective analysis found that those with chronically elevated adolescent-to-adult stress patterns have higher adult cardiometabolic risk.^[33]^ Nevertheless, there is a deficiency in prospective or retrospective studies on the impact of mood or stress on SSS. Future research should concentrate on this area in order to improve the quality of life for SSS patients and lower morbidity and complications. The adults who experienced long-term social isolation had a 1.5-fold increased risk of CVD. At the same time, stress, anger, and excessive sadness are likely to be acute triggers of major cardiovascular events.^[34,35]^

**Figure 1.**
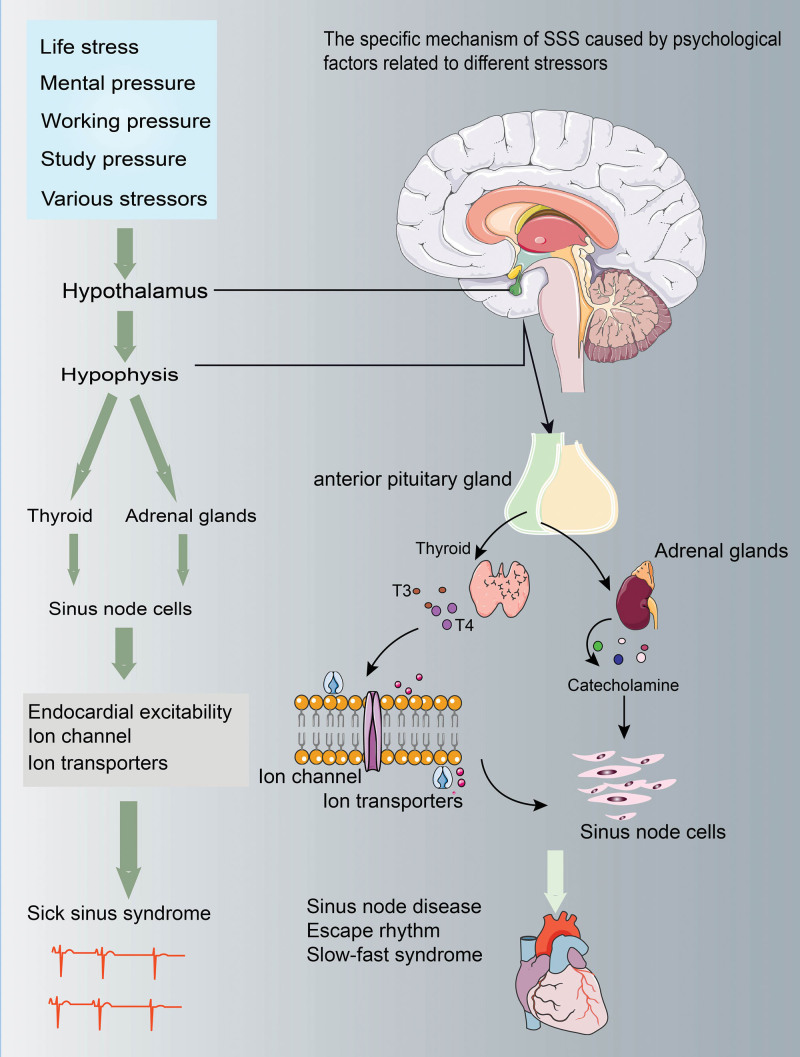
The mechanism of mental factors leading to SSS. A variety of psychiatric factors mediate hypothalamic–pituitary–endocrine system dysfunction, resulting in SSS abnormal heart rate. SSS = sick sinus syndrome.

Although the specific mechanism of how the mental factors associated with different stressors lead to the occurrence of SSS has not been clarified. But for a long time, endocrine and metabolic disorders caused by various mental stimulation may be the pathogenesis of SSS^[36]^ (Fig. 1). During the long-term stress and psychological trauma, thyroid hormone, various neurohormones and target hormones in the lower pituitary gland will produce secretion disorder. However, various hormone regulatory systems play an important role in maintaining normal sinus node function and stabilizing heart rhythm.^[37–39]^ First of all, thyroid hormones and various neurohormones are closely related to the regulation of endocardial excitatory conduction and the maintenance of normal sinus rhythm.^[40]^ Among them, thyroid hormone and various kinds of neurohormones can act on the heart rhythm in many ways, mainly by acting on the ion channel to actively regulate the normal physiological function of sinoatrial node. Therefore, thyroid hormone and all kinds of neurohormone secretion disorder can lead to sinus node and adjacent tissue mucosal degeneration, thus preventing the generation and conduction of sinus rhythm.^[41]^ Secondly, endocrine disorders caused by various stress sources also affect thyroid function. Hyperthyroidism is related to sinus tachycardia or supraventricular tachyarrhythmia accompanied by SSS.^[18]^ The main pathogenesis is that the excessive secretion of thyroid hormone changes the regulation of ion transporter and the action potential produced in pacemaker cells, leading to the formation of abnormal impulses in sinoatrial node. This may be the main pathological mechanism of SSS caused by mental and social stress. In addition, the hypothalamic pituitary adrenal axis also plays a key role in the regulation of rhythm and homeostasis. When the body is under pressure for a long time, the hypothalamic pituitary adrenal axis will be activated, which will eventually lead to the acceleration of heart rate by activating the cardiac sympathetic nervous system and increasing the release of plasma catecholamine.^[42–44]^ The mechanism of this effect will also affect the beating function of sinoatrial node for a long time, leading to the occurrence of SSS and other CVDs.

Further studies have shown that positive emotions are associated with a reduced risk of CVD.^[45]^ The better mood regulation can reduce the risk of CVD among young people to middle-aged community residents. It can be seen that heart health is closely related to pressure and mental factors. People who have been in a stressful and nervous state for a long time should learn to release the pressure and release the bad factors such as anxiety, depression, anger and tension in time, which will contribute to the health of the heart and sinoatrial node.^[46]^ In the future, the main challenge for SSS prevention and treatment is to include negative emotional factors caused by various stressors into the scope of SSS physiological and pathological research.^[47]^

## 3. Excessive drinking

According to a study published by the lancet in 2018, 32.5% of the world’s people are current drinkers (defined as those who drink in the past 12 months).^[48]^ Through this research, we can see that drinking has become a part of many people’s lives. Studies have shown that a small amount of moderate drinking does not affect health and can also reduce the risk of CVD.^[49]^ Limited alcohol intake can reduce the risk of death from CVD, because ethanol can improve the lipid profile and reduce blood coagulation, thus enhancing the protection of the heart.^[50–52]^ So moderate drinking can reduce the risk of CVD. But long-term excessive drinking will aggravate the burden on the heart, increase the oxygen consumption of the heart, lead to arrhythmia and atrial fibrillation.^[53,54]^ In the end, it will form a variety of CVDs such as SSS and aggravate the condition of primary CVDs.^[55–59]^ The pathogenesis of SSS disease caused by excessive drinking may be achieved by the way that the metabolic products of ethanol accelerate the heart rate. Acetaldehyde, a product of ethanol metabolism, can cause the release of norepinephrine in myocardium and promote the release of catecholamine from sympathetic nerve endings.^[53]^ The excessive release of catecholamine will not only lead to the long-term acceleration of heart rate, but also lead to the increase of calcium influx and intracellular calcium concentration in cardiomyocytes.^[42]^ The increase of calcium influx and the concentration of intracellular calcium ions will lead to the membrane potential disorder of cardiac myocytes, which eventually leads to the formation of sinoatrial node block and arrhythmia.^[60]^ This may be the mainly cause of SSS.

In addition, the increased BP caused by excessive drinking may also be involved in the pathogenesis of SSS.^[12,61]^ After drinking too much, the BP of normal people will rise continuously within a few hours. Most studies support that ethanol has a direct effect on the rise of BP.^[62–64]^ Ethanol may inhibit vasodilator and lead to BP rise through abnormal activation of sympathetic nervous system and renin–angiotension system.^[65,66]^ Ethanol may also affect the transport of membrane electrolytes, increase the calcium ion in vascular smooth muscle cells, and lead to the rise of BP.^[66]^ Therefore, the more alcohol intake, the greater the extent of BP rise, and the shorter the duration of BP rise.^[67–69]^ And too much alcohol can also lead to hormone secretion disorder. Excessive or insufficient secretion of all kinds of neurohormones leads to abnormal action potentials in sinoatrial node cells, which eventually leads to the formation of abnormal impulses in sinoatrial node. In the long run, this will lead to the onset of SSS. Therefore, we should pay attention to the negative effects of excessive drinking in the prevention and treatment of SSS. If SSS patients are heavy drinkers, they must be strongly advised to abstain from alcohol, or to significantly reduce alcohol consumption. Regular moderate drinkers should also avoid excessive drinking.^[56]^ In addition, the wine also contains methanol, fusel oil, and other harmful substances. Long term excessive drinking will also cause damage to liver, stomach, pancreas, brain, and other organs, some of which are irreversible.^[70]^ Therefore, the prevention and treatment of cardiovascular and other organ diseases caused by excessive drinking should be studied in the future.

## 4. Smoking

With the progress of modern society and the popularization of medical knowledge, people gradually realize the potential harm of smoking to cardiovascular system. In addition, a large number of clinical and epidemiological studies have found that there is a correlation between smoking and CVD.^[71]^ Harmful substances in tobacco (A. nicotine, B. carbon monoxide, C. aldehydes, nitrides, olefins, D. amines, cyanides, and heavy metals, E. benzopyrene, arsenic, cadmium, methylhydrazine, aminophenol, F. phenolic compounds and formaldehyde, etc) can significantly increase the risk of CVDs.^[72–74]^ Ozdemir^[75]^ found in a retrospective study that long-term smoking increased the risk of arrhythmia, and the index of cardiac electrophysiological balance was also affected. The imbalance of cardiac electrophysiology will eventually lead to the dysfunction of sinoatrial node and the onset of SSS. Nicotine, a harmful substance in cigarette smoke, is an important factor in the increase of smoking addiction and SSS. Nicotine in tobacco is absorbed by alveoli and transported to all parts of the body by blood. At the same time, it can also activate the sympathetic nervous system and increase the release level of catecholamine, which will eventually lead to the acceleration of heart rhythm and the rise of BP.^[76]^ For a long time, it can also affect the normal pacemaker function of sinoatrial node, leading to the onset of SSS. Further studies has reported that the relationship between smoking and CVD that the pathogenesis of smoking leading to acute cardiovascular events may be completed with the participation of nicotine, carbon monoxide, and oxidative stress.^[77]^ A large amount of nicotine absorbed by the blood will accelerate the process of sinoatrial node structural fibrosis, which will lead to the over remodeling of sinoatrial node structure and the appearance of abnormal pulsation of sinoatrial node. Carbon monoxide from smoking also reduces the amount of oxygen carried by red blood cells and is accompanied by excessive oxidative stress.^[78,79]^ Excessive oxidative stress can also increase myocardial stress and decrease ventricular fibrillation threshold. Smoking promotes immune diseases, CVDs and inflammatory diseases by promoting the aggregation and release of inflammatory factors, and inflammation is the basic pathology of arteriosclerosis.^[80]^ So smokers with coronary heart disease are more likely to have arrhythmia, which also affects the physiological function of sinoatrial node. The joint participation of nicotine, carbon monoxide, and oxidative stress will lead to the development of sinoatrial node syndrome or the aggravation of arrhythmia symptoms.

In addition,^[81–83]^ during smoking, the plasma levels of adrenaline, β-blood cell protein, and fibrinogen, which are important components of the coagulation system, will rise to different degrees. Fibrinogen is an auxiliary factor of platelet aggregation. The increase of fibrinogen will accelerate the process of atrial and sinoatrial node fibrosis, lead to the occurrence of sinoatrial node remodeling and induce SSS.^[84]^ Carbon monoxide in tobacco smoke combines with hemoglobin, reducing the oxygen carrying capacity of hemoglobin, and preventing hemoglobin from releasing oxygen. This anoxia process can lead to the remodeling of sinoatrial node tissue and the dysfunction of sinoatrial node pulsation.^[82,85]^ For the patients with CVD, the increase of carbon monoxide caused by smoking can also cause ventricular arrhythmia, leading to the aggravation of the primary disease.^[86,87]^ So quitting smoking is beneficial to reduce the prevalence of SSS, and patients with SSS should also be discouraged from smoking to slow down the disease.^[88]^ How to promote the policy of smoking ban will also become one of the important measures to reduce the incidence rate of SSS. Smoking also causes respiratory injury and cerebrovascular disease. The longer the smoking volume and the longer the smoking period, the higher the incidence rate and mortality rate of cardiovascular, cerebrovascular and respiratory system. Moreover, smoking can cause damage to people’s health as well as damage to themselves.^[88,89]^ Second hand smoke, for example, can quickly damage the heart and brain vessels, leading to an increased risk of stroke and coronary heart disease by 25% to 30%. So short term exposure to secondhand smoke can also lead to an acute attack of CVD.^[90–92]^ So quitting smoking at any age can benefit cardiovascular and other organs.

## 5. Unhealthy diet

Diet plays a count for much role in people’s daily life. Maintaining healthy eating habits is very important for cardiovascular health.^[93–95]^ In recent years, unhealthy eating habits have been included in the risk factors of CVD, and can directly affect the pathogenesis of SSS. Unhealthy eating habits (high fat, high salt, high sugar, high cholesterol, etc) affect triglycerides, cholesterol, BP, hormone secretion, and other indicators of the human body. The above indicators and the high BMI caused by unhealthy diet are also one of the reasons for the high prevalence of SSS.^[12,96]^ Therefore, how to maintain a reasonable diet to reduce the incidence of SSS and further reduce the incidence rate and mortality of CVDs is a problem to be solved urgently. It is worth noting that reasonable control of fat, calories, salt, and sugar intake can reduce the incidence rate of CVDs and SSS, and now it has become a consensus in the medical field. This consensus has been written into healthy dietary guidelines and CVD prevention guidelines.^[97–99]^

Among the unhealthy eating habits, high-fat diet is one of the most harmful to cardiovascular system. Studies have reported that obesity caused by high-fat diet is an important risk factor for ventricular arrhythmia. High fat diet can lead to left ventricular hypertrophy and fibrosis, and reduce the protein expression of L-type calcium channel Cav1.2.^[100]^ The abnormal expression of calcium channel Cav1.2 will further reduce the self-regulation of sinoatrial node cells and lead to sinoatrial node dysfunction. Therefore, this study confirmed that the incidence of sinoatrial syndrome is closely related to high-fat diet, for example, obesity and dyslipidemia caused by high-fat diet are important risk factors of sinoatrial syndrome. Further research^[101]^ also found that the mice fed with high-fat diet showed prolonged P-wave duration and induced increase of sustained tachycardia through arrhythmia study of adult male mice fed with high-fat diet. In addition, the expression of inflammatory cytokines in the blood of mice fed with high-fat diet also increased significantly. At the same time, the over expression of inflammatory cytokines will affect many kinds of ion channels, which will eventually lead to the disorder of sinus node electrophysiological activity and promote the occurrence of arrhythmia. In addition, the increase of BP caused by high salt diet and insulin level caused by high sugar diet are also problems worthy of attention in the process of SSS prevention. Clearly, a healthy diet rich in fruits, vegetables, and nuts is advantageous. For instance, cardiovascular and metabolic risk factors like BP, lipids, blood sugar, obesity, and inflammatory markers are all improved by a diet high in pistachios.^[102]^. In the future, clinical nutrition and metabolic research may make use of visceral adipose tissue, which is an accurate predictor of death from CVD.^[103]^

It is imperative that people adopt a healthy diet, with a focus on low-fat lifestyle measures. The Mediterranean Diet (MED) is a widely recognized strategy for the primary and secondary prevention of CVD, and it has been shown to considerably lower the risk of CVD.^[104–107]^ Consuming a wide variety of fruits, vegetables, and grains is the most essential aspect of the MED. One of the key benefits of the MED is that it creates a synergistic impact between a wide range of foods and elements that are cardioprotective.^[108–110]^ These foods are particularly rich in plant steroids and dietary fiber, and they offer a variety of minerals, vitamins, and antioxidants.^[104]^ MED reduces the risk of SSS by lowering BP and lipids, increasing nitric oxide bioavailability, antioxidant and anti-inflammatory effects. The dietary approaches to stop hypertension (DASH) reduces the risk of CVD precisely by effectively lowering systolic blood pressure (SBP).^[111]^ The DASH diet places a strong emphasis on a “combination diet” that consists mostly of fruits, vegetables, low-fat dairy products, whole grains, chicken, fish, and nuts. In contrast to the usual American diet, it is low in sugar, sugar-containing foods, and high in dietary fiber and protein.^[112]^ Prehypertension and SBP were inversely correlated with DASH and MED diet adherence.^[113]^

Mediterranean food structure can reduce the risk of SSS by reducing BP and lipids, increasing the bioavailability of nitric oxide, anti-oxidation, and anti-inflammatory effects. It should be noted that the Mediterranean diet also advocates moderate consumption of red wine in meals, which is in line with the above mentioned moderate drinking recommendations. Therefore, healthy eating habits are important to reduce the risk of SSS and maintain heart health.

In addition, the ketogenic diet is not a minority of advocates. Strictly speaking, the ketogenic diet does not meet the criteria for a healthy diet, although it may provide rapid short-term reductions in body weight, triglyceride levels, and BP. However, in terms of long-term benefits, the ketogenic diet is not effective in reducing fat and improving glucolipid metabolism.^[114]^ However, there is very little evidence on the ketogenic diet in comparison with the Mediterranean diet. Even when compared with a very low carbohydrate intake, such as the ketogenic diet, reasonable control of carbohydrate intake and maintaining a low carbohydrate pattern over time is more effective in reducing cardiovascular mortality.^[115]^ Adverse cardiovascular events associated with ketosis is a direction that future studies must take into consideration.

## 6. Sedentary and lack of exercise

With the rapid development of modern science and technology, people’s daily life and commuting mode have been changed by changing lifestyle and means of transportation, which has led to a significant decline in people’s daily activity level. According to the latest investigation and research,^[116–118]^ sedentary and long-term inactivity have become the living habits of many indoor workers and the elderly.^[30]^ It is worth noting that people who do not exercise for a long time are likely to be in a sub-health state.^[76]^ Once further developed, they are likely to suffer from CVD.^[119]^ It has been found that sedentary and lack of exercise lifestyle can lead to obesity, high BP, SSS, and a variety of CVDs, threatening people’s health.^[120,121]^ The recent meta-analysis study has been reported that only high levels of moderate to vigorous physical activity of 60 to 75 minutes per day could reduce the association between total sedentary time and all-cause mortality.^[122]^ In addition, long-term inactivity of watching TV and computer will bring death risk of CVD.

Although how lack of exercise is involved in the pathogenesis of SSS has not been explained clearly, it is likely to be related to lack of exercise and sedentary BP rise. Wendy^[123]^ found a positive correlation between sedentary lack of exercise and SBP in a study of the relationship between sedentary time and cardiac metabolic health in obese adults. The risk of high BP increased by 14% for each hour of sitting posture increase. Beunza^[124]^ tracked the incidence of hypertension in 6742 healthy college graduates over a 40 month period in a prospective, dynamic cohort study. The study found that sedentary subjects had a 48% increased risk of high BP compared with their exercise-friendly counterparts. Lee^[125]^ found a certain correlation between baseline and time changes of physical activity and sudden hypertension or diabetes. Enough baseline physical exercise can significantly reduce the risk of hypertension. With the increase of exercise time, the risk of hypertension is significantly reduced.

The pathogenesis of arrhythmia and sinoatrial node function caused by sedentary and lack of exercise may be due to the over secretion or under secretion of various neurohormones caused by hypertension, which affects the electrophysiological balance of sinoatrial node cells. Sinoatrial node cells produce abnormal action potential, which leads to dysfunction of pacing function and abnormal impulse formation. The increase of BP will also lead to the increase of calcium concentration in the body. The increase of calcium concentration is the second phase of calcium influx in the process of myocardial depolarization, which will increase the intracellular calcium concentration.^[126]^At the same time, it improves the contractility and oxygen consumption of the heart muscle, increases the self-regulation of the sinoatrial node cells, accelerates the atrioventricular conduction, and finally leads to the occurrence of arrhythmia and SSS. The increase of BP caused by lack of exercise and sedentary will also destroy normal myocardial fiber tissue and slow down conduction, generate electrical heterogeneity, and lead to abnormal electrical activity, which is also the potential mechanism of SSS.^[126,127]^ It can be seen that proper exercise of human body is closely related to physical health,^[128]^ moderate exercise can prevent CVDs such as SSS. In addition to moderate to vigorous exercise and maintaining a healthy weight, prolonged sitting should be avoided, especially among the elderly and those who work indoors.

## 7. Sleep deprivation and poor sleep quality

In the process of prevention and treatment of SSS and many CVDs, the importance of ensuring good sleep time and sleep quality has been widely recognized by the medical community.^[129–131]^

With the rapid development of society and the increase of work pressure, people’s sleep time is gradually reduced.^[132]^ According to a survey of people who sleep less in the United States,^[133]^ people who sleep <6 hours a night tend to work longer. In 1975 to 2006, the survey found that the sleep time of full-time workers decreased significantly with the development of society. The percentage of people who sleep <6 hours per night on working days is also increasing year by year. According to the National Sleep Foundation report, only 44% of Americans say they have high sleep quality every night.^[134]^ Short sleep time or poor quality can also cause and aggravate many CVDs such as myocardial infarction, heart failure, coronary heart disease, and SSS.^[107]^ In many patients with CVDs, the proportion of patients with sleep disorders is very high. CVD patients often have arrhythmia or even sudden death during sleep. SSS disease, short sleep duration, and sleep quality affect each other, so it is very important to pay attention to the sleep situation of SSS patients.

At present, the research of sleep affecting nervous system and indirectly destroying cardiovascular system leading to SSS has been widely carried out. Wolk^[135]^ have shown that too short sleep time can increase the excitability of sympathetic nerve and decrease the excitability of vagus nerve. The balance between sympathetic and vagus nerves of the heart is broken, which eventually leads to a serious reduction of heart rate variability (HRV). The fatal arrhythmia is related to the increase of sympathetic nerve excitability and the decrease of vagus nerve excitability, because the balance of them directly affects the HRV. HRV refers to the small difference in the interval between successive heart beats. It arises from the modulation of the autonomic nervous system on the sinoatrial node of the heart, which causes the difference and fluctuation of the interval between heart beats in tens of milliseconds. HRV is essentially a reflection of the regulatory effect of neurohumoral factors on sinoatrial node. When the balance between sympathetic activity and vagal activity of autonomic nervous system is broken, the normal physiological function of sinoatrial node will be destroyed. If the abnormal HRV decreases, the arrhythmia caused by the abnormal impulse of sinoatrial node will appear, which will cause the onset of SSS.

In addition, the increase of BP caused by sleep deprivation and poor sleep quality may also be involved in the mechanism of SSS. It is known that the secretion system of neurohormone is closely related to the physiological rhythm of sleep. Norepinephrine and adrenaline in the plasma of normal people reach the highest level in the morning and the lowest level in the night. Sleep deprivation and poor sleep quality can lead to the imbalance of norepinephrine and adrenaline secretion, and this process will directly affect the renin–angiotension–aldosterone system, pituitary adrenal system and hypothalamus pituitary thyroid system, and eventually lead to the rise of BP.^[136]^ The long-term increase of BP will indirectly lead to the imbalance of calcium concentration inside and outside the cardiac myocytes and sinoatrial node cells, and eventually lead to the autonomic disorder of sinoatrial node cells and destroy the normal physiological function of sinoatrial node. In addition, sleep apnea syndrome (obstructive sleep apnea hypopnea syndrome [OSAHS]) has become the main cause of CVD.^[137,138]^ Oral microflora (actinomycetes, *Porphyromonas gingivalis*, *Prevotella intermedia*, etc) caused by sleep apnea syndrome may play a pathological role in many ways. Oral microflora can lead to platelet aggregation, immune response, inflammatory response and oxidative stress in human body, and ultimately lead to sinus node dysfunction and SSS in OSAHS patients. Although sleep deprivation and poor sleep quality, and how sleep apnea syndrome (OSAHS) is involved in the pathogenesis of SSS have not been elucidated. However, patients who lack of sleep time should adjust their work and rest time in time to avoid heart overload. Patients with insomnia and OSAHS should be diagnosed and treated in time to reduce the risk of SSS and other CVDs. Future research should also further study and reveal the relationship between sleep and the risk of SSS, and bring new methods and ideas for the treatment of this disease.

## 8. Discussion

CVD development is influenced by the gut microbiota. Modifications to the intestinal flora’s makeup and ecology (known as gut dysbiosis). Examining SSS from gut flora could be a novel approach for further study. Ingested food is metabolized by gut flora producing a variety of metabolites, such as indoxyl sulfate, trimethylamine N-oxide, short chain fatty acids, and secondary bile acid, which impact the development of CVD by activating several signaling pathways.^[139]^ Although genetic and environmental variables play a major role in the characterization of the gut microbiota. Nonetheless, nutrition is a major factor in controlling the gut microbiota’s makeup. A nutritious diet influences fermentation metabolism, gut pH, and the proliferation of particular bacterial strains, all of which can impact the formation of pathogenic flora. A diet high in fat promotes the growth of a gut microbiota that is proinflammatory, increasing intestinal permeability and lipopolysaccharide levels in the blood.^[140]^ Therefore, by optimizing the gut flora through food planning, the incidence of CVD can be decreased.^[141]^ In terms of the metabolic alterations in the gut microbiota, regular exercise is also advantageous.^[142]^ Sleep patterns also disrupt the gut microbiota and metabolic rhythms, supporting the notion of the microbiota–gut–brain axis.^[143]^

Because the pathogenesis of SSS is extensive and the pathological mechanism is complex, how to participate in the pathogenesis of SSS and the interaction of multiple mechanisms should be included in clinical and experimental research. At present, due to the limitations of the survey population and the coverage of the research results, some limitations of this study must be considered. So there are many factors and conditions to be considered in the future research.

First of all, the main purpose of current epidemiological survey is to study the patients with SSS and high-risk population, and there are many patients with coronary heart disease combined with SSS and other CVDs accompanied with SSS. Therefore, in the future, the study on the relationship between adverse lifestyle and SSS should distinguish the patients with SSS and those with other CVDs. Second, the research direction should not only observe the pathogenesis of sinoatrial node dysfunction, but also explore the negative factors of bad lifestyle on other CVDs. We should also study the interaction mechanism between SSS and other complications under the influence of adverse lifestyle and the interaction between adverse lifestyle.^[144]^Third, although some SSS patients with pacemaker have been covered, there is no targeted lifestyle study on SSS patients with pacemaker. In the future, we can also include the study of the influence of bad lifestyle on patients with SSS who have already implanted pacemaker, so as to guide the lifestyle of patients with SSS who have already implanted pacemaker. Fourth, although some experimental studies have elucidated the mechanism of multiple bad lifestyles in the pathogenesis of SSS. However, because of the complexity of the pathogenesis of SSS and the uncertainty of the study, there are too many factors. In the future, we should carefully consider the two-way causal relationship between bad lifestyle and SSS, and conduct targeted prospective research. Fifth, because the instability of living habits is affected by age, region, working environment, living environment and other factors, the results of a single survey may not necessarily reflect the long-term lifestyle of the personnel. Therefore, it is necessary to make a detailed follow-up plan and follow-up for many times in the study to get reliable research results. Sixthly, there are large differences in age and gender in some clinical and epidemiological studies. For example, whether the physiological degenerative process of sinoatrial node fibrosis in the elderly and the pathological process of sinoatrial node physiological dysfunction caused by poor lifestyle in the young should be reasonably distinguished, they are likely to be included in the same investigation and study together. In addition, with the difference of age and gender, the hormone secretion system and neural regulatory system of human body are also different. These factors will have a certain impact on the research results.

It is evident that the aforementioned restrictions are crucial to expanding the research on the connection between a low lifestyle and SSS. Future research can examine the impact of medical conditions and the environment on the microbial structure from the perspective of the gut flora in order to develop more thorough and effective preventative and treatment protocols. Additionally, it offers scientific support for clinical diagnosis and treatment of CVDs.

## 9. Conclusion

To sum up, This study found that SSS is closely related to mental stimulation and various unhealthy lifestyles. As shown in Table 1, mental factors, unhealthy diet, smoking, drinking and lack of exercise caused by various stressors may induce or aggravate SSS. However, maintaining a good lifestyle can reduce the risk of SSS, and help SSS patients to control the disease well. Although many epidemiological and pathogenesis studies have shown that unhealthy lifestyle is related to the pathogenesis of SSS.As shown in Table 2, it is worth noting that personal behaviors such as drinking, lack of exercise and mental factors do not directly damage the physiological function of sinoatrial node. But it leads to the physiological dysfunction of sinoatrial node by increasing hypertension and regulating the secretion system of neurohormone abnormally. This is very important for the prevention and treatment of healthy people and SSS patients.

**Table 1:** Adverse factors leading to SSS.

Classification	
Pressure source	A. Physical stressor, B. social stressor, C. cultural stressor , D. psychological stressors
Unhealthy emotions	A. Anxiety, B. depression, C. fear, D. anger , E. indifference, F. tension, G. sadness
Unhealthy eating habits	A. High fat diet, B. high cholesterol, C. high calorie , D. less intake of vegetables and fruits, E. less intake of grains, F. high salt and sugar
Unhealthy sleep habits	A. Insufficient sleep time, B. poor sleep quality, C. obstructive sleep apnea hypopnea syndrome (OSAHS)
Smoke	A. Nicotine, B. carbon monoxide, C. aldehydes, nitrides, alkenes , D. amines, cyanides and heavy metals, E. benzopyrene, arsenic, cadmium, methylhydrazine, aminophenol, F. phenolic compounds and formaldehyde
Alcohol	A. Acetaldehyde, B. methanol, C. fusel oil
Lack of exercise	A. Sedentary, B. long term indoor work

**Table 2:** The pathogenesis of SSS caused by unhealthy life factors.

Classification	Indirect pathological mechanism	Effect on physiological function of sinoatrial node
Unhealthy emotions	(1) Thyroid hormone secretion disorders. (2) Pituitary and downstream target hormone secretion disorders.^[40–42]^ (3) Maladjustment of humoral regulation system and secretion of neurohormone.^[39]^	(1) Mucosal degeneration of sinoatrial node and adjacent tissues. (2) Production and conduction of sinus rhythm.^[39]^ (3) Regulation of ion transporters on sinoatrial node cells.^[38]^
Unhealthy diet	(1) Left ventricular hypertrophy and fibrosis, protein expression of L-type calcium channel Cav1.2 decreased.^[97]^ (2) The induction of sustained tachycardia was increased, and the expression of inflammatory cytokines was significantly increased.^[98]^	(1) The self-discipline of sinoatrial node cells is decreased. (2) The electrophysiological activity of sinoatrial node is disordered.^[98]^
Unhealthy sleep habits	(1) The excitability of sympathetic nerve increased, but that of vagus decreased.^[25]^ (2) The variability of heart rate decreased. (3) Renin-a-aldosterone system disorder, pituitary adrenal system disorder, hypothalamus pituitary thyroid system disorder, blood pressure rise leads to calcium concentration imbalance.^[126]^ (4) Oral microbial changes induce inflammation, oxidative stress and so on.^[127,128]^	(1) The normal physiological function of sinoatrial node will be destroyed. (2) Decreased abnormal heart rate variability, arrhythmia caused by abnormal impulse of sinoatrial node.^[125]^
Smoke	(1) Cardiac electrophysiological balance, (2) index (iCEB) decreased, catecholamine release increased, (3) excessive oxidative stress, (4) myocardial stress increased, (5) ventricular fibrillation threshold decreased.^[74]^ (6) Increased levels of adrenaline,β- hemoglobin and fibrinogen.^[78–83]^	It leads to fibrosis of sinoatrial node structure, cause sinoatrial node remodeling.^[71,81–83]^
Alcohol	(1) Excessive release of noradrenaline and catecholamine.^[51]^ (2) Calcium influx and membrane potential abnormality in cardiomyocytes,^[40]^ elevated blood pressure.^[12,59]^ (3) Activate sympathetic nervous system and renin-a system, inhibit vasodilator. (4) Calcium or other electrolytes in vascular smooth muscle cells increased. (5) Neurohormone secretion disorder, cardiac pacemaker cells produce abnormal action potential.^[60–62]^	Sinoatrial node block and arrhythmia.^[60]^ Abnormal action potential in sinoatrial node cells.^[54]^
Lack of exercise	(1) Neurohormone secretion disorder. (2) Cardiac pacemaker cells produce abnormal action potential. (3) Sinoatrial node electrophysiology disorder and abnormal impulse, body calcium concentration increases, and intracellular calcium concentration increases.^[111]^ (4) Myocardial contractility and oxygen consumption increase, and normal myocardial fiber tissue is destroyed, resulting in electrical heterogeneity^[116,117]^	The self-regulation of sinoatrial node cells is increased, the atrioventricular conduction is accelerated, and the electrophysiological function of sinoatrial node is abnormal^[116,117]^

At present, the treatment of SSS in the world takes up a lot of social medical and health resources, and causes a huge burden on families and social economy. With the aging and urbanization, the prevalence of lifestyle risk factors has not been effectively curbed. We should mobilize all social forces to participate widely, improve the public’s health literacy level and active health awareness. In combination with new technologies and new ways such as mobile medical and wearable technology, we should focus on strengthening scientific guidance of lifestyle such as reasonable diet, moderate increase in physical activity, maintaining healthy weight, smoking cessation and alcohol restriction. Promote the popularization of healthy lifestyle in the general population, especially in the patients with SSS. This will be an important way to improve the cardiovascular health status of residents and help to promote the specific implementation of global health action. Ultimately, it will reduce the burden of disease of the whole society and patients, and improve the health level.

## Acknowledgments

I am profoundly grateful to my supervisor, Ruxiu Liu, whose illuminating instruction and expert advice have guided me through every step of my writing of this thesis.

## Author contributions

**Data curation:** Yanli Wang.

**Formal analysis:** Jinfeng Liu, Xuanke Guan, Zhiming Liu.

**Investigation:** Siyuan Zhou.

**Methodology:** Qiaomin Wu, Zhiming Liu, Ruxiu Liu.

**Supervision:** Zhiming Liu, Ruxiu Liu.

**Validation:** Ruxiu Liu.

**Writing – original draft:** Xing Chang, Xiangyi Pu.

**Writing – review & editing:** Xing Chang, Qin Zhang.
